# Long-term surgical outcome of high myopic full-thickness macular hole without retinoschisis

**DOI:** 10.1186/s12886-025-04325-z

**Published:** 2025-08-25

**Authors:** Sung Do Cho, Eun Kyoung Lee, Chang Ki Yoon, Un Chul Park

**Affiliations:** 1 Department of Ophthalmology, Seoul National University Hospital, Seoul, Korea; 2 Department of Ophthalmology, Seoul National University College of Medicine, Seoul, Korea; 3 Department of Conscription Examination, Seoul Regional Office of Military Manpower, Seoul, Korea

**Keywords:** Myopia traction maculopathy, Macular hole, Optical coherence tomography parameters, Surgical outcome

## Abstract

**Background:**

To examine the clinical features and long-term surgical results of highly myopic full-thickness macular hole (FTMH) without retinoschisis.

**Methods:**

We retrospectively assessed surgical outcomes of highly myopic patients who underwent vitrectomy with internal limiting membrane peeling and gas tamponade to treat FTMH without retinoschisis. Post-operative examinations were conducted at 1, 3, 6, and 12 months after surgery, with further follow-up intervals adjusted based on physician’s discretion. Clinical features related to visual acuity and anatomical success, defined as hole closure without developing foveal atrophy, were assessed.

**Results:**

Thirty-three eyes (30 patients) were included, and the mean follow-up period was 61.1 ± 48.8 months. Macular hole closure was achieved in 31 (93.9%) eyes, of which 16 (51.6%) developed foveal atrophy during follow-up. Eyes with anatomical success exhibited a significant better visual prognosis than those without during whole follow-up period (*P* ≤ 0.031), showing a significant postoperative visual improvement from baseline at 6 months, 12 months and final visit (*P* = 0.002, *P* < 0.001 and *P* = 0.013, respectively). Greater minimal macular hole diameter (*P* = 0.002) and the presence of posterior staphyloma (*P* = 0.040) were significantly related to anatomical failure, whereas longer axial length was significantly related to poorer final best-corrected visual acuity (*P* < 0.001).

**Conclusions:**

Hole closure rate after vitrectomy in highly myopic patients with FTMH without retinoschisis was high. However, the postoperative development of foveal atrophy hindered vision improvement. Posterior staphyloma, greater minimal hole diameter, and longer axial length were predictive of poor prognosis.

## Background

High myopia is one of the leading etiologies of permanent visual loss globally, especially in East Asia, where the prevalence of myopia is increasing rapidly [1, 2]. Visual loss in high myopia is typically caused by pathological changes in the macula, including chorioretinal atrophy, myopic choroidal neovascularization (mCNV), and myopic traction maculopathy (MTM) [3]. In the clinical course of MTM, structural changes in the posterior eyeball can cause anterior-posterior and tangential traction, resulting in foveoschisis progressing into foveal detachment, full-thickness macular hole (FTMH), and ultimately macular hole retinal detachment. The clinical features of foveoschisis-induced FTMH in high myopia differ from those of idiopathic FTMH in non-myopic eyes with respect to configuration on optical coherence tomography (OCT), pathophysiology, and postoperative closure rate [4–7].

Although FTMH with high myopia generally manifests as a part of MTM involving retinoschitic change, another subgroup of FTMH occurs without myopic retinoschisis. Compared to FTMH associated with myopic retinoschisis, FTMH without retinoschisis tends to occur in eyes with a less severe phenotype of high myopia, showing better anatomical and functional results after pars plana vitrectomy (PPV) combined with internal limiting membrane (ILM) peeling and gas tamponade, the current standard surgical treatment for FTMH [5, 8, 9]. However, the long-term surgical outcomes of non-schitic FTMH in highly myopic eyes remain unclear. Additionally, recent findings in highly myopic eyes suggest that the development of fovea-centered macular atrophy following PPV for MTM may impair functional recovery, even when hole closure is successful in non-schitic FTMH [10]. We aim to assess the clinical features and long-term surgical results of highly myopic FTMH without retinoschisis and to determine prognostic factors related to postoperative anatomical and functional outcomes.

## Methods

### Patients

In this retrospective study, we reviewed the electric medical records of patients with high myopia who underwent vitrectomy to treat FTMH without myopic retinoschisis between January 2008 and April 2022 at the Seoul National University Hospital (SNUH). High myopia was characterized by an axial length (AXL) of at least 26.0 mm or a myopic refractive error of −6.0 diopters or greater. The exclusion criteria included: (1) presence of any outer retinoschitic change on OCT imaging; (2) history of ocular trauma or previous vitreoretinal surgery; (3) macular pathologic conditions unrelated to high myopia that could cause visual loss; (4) postoperative follow-up of < 1 year; and (5) pre-existing foveal atrophy (FA), which was defined as atrophy involving the area of a 1.5 mm diameter circle centered on the foveal center. The presence of intraretinal cystic changes, elevated cuff at the FTMH margin, and inner retinoschitic change that did not involve the FTMH margin were not regarded as exclusion criteria.

The Institutional Review Board of SNUH approved this study (IRB No.2410-009-1574), and the research was conducted in accordance with the tenets of the Declaration of Helsinki. Due to the retrospective design of the study, informed consent was waived.

### Examinations

All the included participants had a comprehensive ophthalmic evaluation, which comprised best-corrected visual acuity (BCVA) assessment, measurement of refractive error and intraocular pressure, slit-lamp examination, dilated fundus examination, AXL measurement (IOL Master 700, Carl Zeiss Meditec, Jena, Germany), spectral domain OCT (Spectralis OCT; Heidelberg Engineering, Heidelberg, Germany), and ultra-widefield retinal imaging (Optos 200Tx; Optos PLC, Dunfermline, UK). BCVA was converted to the logarithm of the minimal angle of resolution (logMAR) values for statistical analysis. Visual acuities for light perception, hand movement, and counting fingers were assigned logMAR values of 3, 2.5, and 2, respectively. Macular hole parameters, including minimal diameter, basal diameter, macular hole height, hole form factor (HFF), tractional hole index (THI), macular hole index (MHI), and diameter hole index (DHI), were manually measured using a caliper on the OCT machine [11–13]. The minimal diameter referred to the narrowest distance across the hole measured at the level of the inner retina, whereas the basal diameter was defined as the widest distance between the edges of the FTMH at the level of the retinal pigment epithelium (RPE) [11]. The macular hole height was defined as the maximum vertical distance from the RPE to the ILM on the steeper side of the hole [11]. The HFF was defined as the sum of the distances from each end of the basal diameter to the corresponding ends of the minimal diameter, divided by the basal diameter [11]. The MHI was calculated by dividing the macular hole height by its basal diameter [13]. The DHI was defined as the ratio of the minimal diameter to the basal diameter, while the THI was obtained by dividing the macular hole height by the minimal diameter [12]. Measurements of horizontal and vertical B-scan OCT imaging were averaged for each parameter. Additionally, other features observed on OCT B-scan images, such as the presence of epiretinal membrane (ERM), dome-shaped macula (DSM), elevated cuff, cystic change at the hole margin, and vitreomacular traction, were evaluated. A DSM was characterized by an inward bulging of the macular RPE by ≥ 50 μm above the estimated line of the RPE at the base of the macular curvature [14]. Posterior staphyloma (PS) was detected through ultra-widefield retinal imaging and classified based on the Ohno-Matsui classification: wide macular, narrow macular, peripapillary, nasal, inferior, and others [15, 16]. Furthermore, myopic maculopathy was categorized using ultra-widefield retinal images and OCT, following ATN classification, where atrophy grade included A1 (tessellated fundus only), A2 (diffuse chorioretinal atrophy), A3 (patchy chorioretinal atrophy), and A4 (macular atrophy) and neovascular grade includes N0 (no mCNV), N1 (macular lacquer cracks), N2a (active CNV), and N2s (scar or fuchs spot) [3].

### Surgical techniques

A standard 23- or 25-G three-port PPV (Constellation Alcon Vision System LXT, Alcon Laboratories, Fort Worth, TX) was carried out by one of five experienced retinal surgeons (Eun Kyoung Lee, Jang Won Heo, Hum Chung, Hyeong Gon Yu and Un Chul Park) with combined phacoemulsification and intraocular lens implantation in eyes with considerable lens opacity. Following core vitrectomy, the posterior vitreous detachment was evaluated using triamcinolone acetonide, and any residual vitreous cortex over the retina was carefully removed. The ILM was stained using 0.05% indocyanine green (ICG) dye diluted with 5% dextrose for 10 s and peeled up to the vascular arcades. Gas tamponade was done with 18% sulfur hexafluoride (SF6) or 14% perfluoropropane (C3F8). Patients were guided to maintain a head-down position for 3–5 days. All participants were given standard postoperative care with topical antibiotics and anti-inflammatory medications. Follow-up evaluations, including BCVA, intraocular pressure measurement, slit-lamp examination, dilated fundus examination, and spectral domain OCT, were conducted at 1, 3, 6, and 12 months after surgery, with further follow-up intervals adjusted based on physician’s discretion. In eyes that failed to achieve FTMH closure after the first surgery, additional surgery was carried out at the surgeon’s discretion. At the final evaluation, anatomical success was characterized by FTMH closure without the postoperative development of FA, which was determined by focal white-yellowish lesion on the fovea in the fundus photographs showing hyperautofluorescence on fundus autofluorescence imaging or increased light penetrance at the fovea on OCT B-scan imaging. Anatomical failure was defined as failure to achieve FTMH closure or development of FA after successful FTMH closure following PPV. Moreover, the timing of the development of postoperative FA was recorded.

### Statistical analysis

Two independent graders (EKL and CKY) measured macular hole parameters and graded macular findings based on the definitions outlined above. Any discrepancies were resolved by a senior retinal specialist (UCP), and the macular hole parameter values from the two graders were averaged for statistical analysis. Univariate and multivariate analyses were conducted to investigate factors related to anatomical failure and final BCVA, respectively. Variables with a *P* value less than 0.1 in univariate analysis were included in the multivariate analysis. The Cox proportional hazard model was applied to assess anatomical failure, with odds ratios (ORs) along with their 95% confidence intervals (CIs). Linear regression analysis was used for the final BCVA. A *P* value of less than 0.05 was considered statistically significant. SPSS software version 22.0 (SPSS, Inc., Chicago, IL, USA) was used for statistical analysis.

## Results

### Population

A total of 33 eyes from 30 patients (four males and 26 females) were included in this study. All patients underwent intraoperative ILM peeling and gas tamponade, and concomitant cataract surgery was carried out in six (18.2%) eyes. Baseline features of the participants are summarized in Table 1. Mean age at surgery was 64.9 ± 8.9 years (range: 49–78 years). Mean AXL and preoperative logMAR BCVA were 30.66 ± 2.11 mm (range: 25.95–35.10 mm) and 0.72 ± 0.38 (range: 0.10–2.00), respectively. Mean duration of preoperative FTMH and follow-up were 5.3 ± 17.3 (range: 0–101 months) and 61.1 ± 48.8 months (range: 12.0–171.0), respectively. Two eyes had a prior history of mCNV (ATN classification N2a) which had stabilized after three monthly injections and a single intravitreal injection of bevacizumab, respectively. The mCNV remained inactive throughout the entire follow-up period, including the time of surgery.

**Table 1:** Baseline demographics and ophthalmic conditions of study participants

Variables	*N* (%)	Mean ± SD	Range
Demographics			
Participants/eyes	30/33		
Age (years)		64.91 ± 8.90	49–78
Gender (male/female)	6/27		
Ophthalmic conditions			
Laterality (L/R)	15/18		
Axial length (mm)		30.66 ± 2.11	26.15–35.10
Lens status			
Phakia	6 (18.2%)		
Pseudophakia	22 (66.7%)		
Aphakia	5 (15.1%)		
Preoperative logMAR BCVA		0.72 ± 0.38	0.10-2.00
MH duration (months)		5.26 ± 17.34	0-101
ATN classification (Atrophy)			
1	8 (24.2%)		
2	12 (36.4%)		
3	13 (39.4%)		
4	0 (0%)		
ATN classification (Neovascular)			
0	26 (78.8%)		
1	0 (0%)		
2a	2 (6.1%)		
2s	5 (15.1%)		
Epiretinal membrane	24 (72.7%)		
Dome shaped macula	17 (51.5%)		
Intraretinal fluid	29 (87.9%)		
Cuff	30 (90.9%)		
MH margin cystic change	26 (78.8%)		
Vitreous traction	8 (24.2%)		
Previous Anti-VEGF	2 (6.0%)		
Posterior staphyloma	26 (78.8%)		
Wide macular	22 (66.7%)		
Narrow macular	3 (9.1%)		
Peripapillary	1 (3.0%)		
Nasal	0 (0%)		
Inferior	0 (0%)		
Others	0 (0%)		
Macular hole indices			
Mean minimal diameter (µm)		324.67 ± 147.34	97.50-662.50
Mean basal diameter (µm)		711.49 ± 242.71	302.0-1622.5
Mean macular hole height (µm)		306.74 ± 81.04	151.0-464.0
Mean HFF		0.82 ± 0.27	0.26–1.93
Mean THI		1.29 ± 1.05	0.22–6.07
Mean MHI		0.49 ± 0.21	0.17–1.16
Mean DHI		0.47 ± 0.19	0.14–1.20
Follow-up (months)		61.18 ± 48.83	12–171

*N* numbers, *SD* Standard deviation, *S.E.* Spherical equivalent, *BCVA* Best corrected visual acuity, *logMAR* Logarithm of the minimum angle of resolution, *MH* Macular hole, *VEGF* Vascular endothelial growth factor, *HFF* Hole form factor, *THI* Tractional hole index, *MHI* Macular hole index, *DHI* Diameter hole index

### Anatomical results

**Fig. 1:**
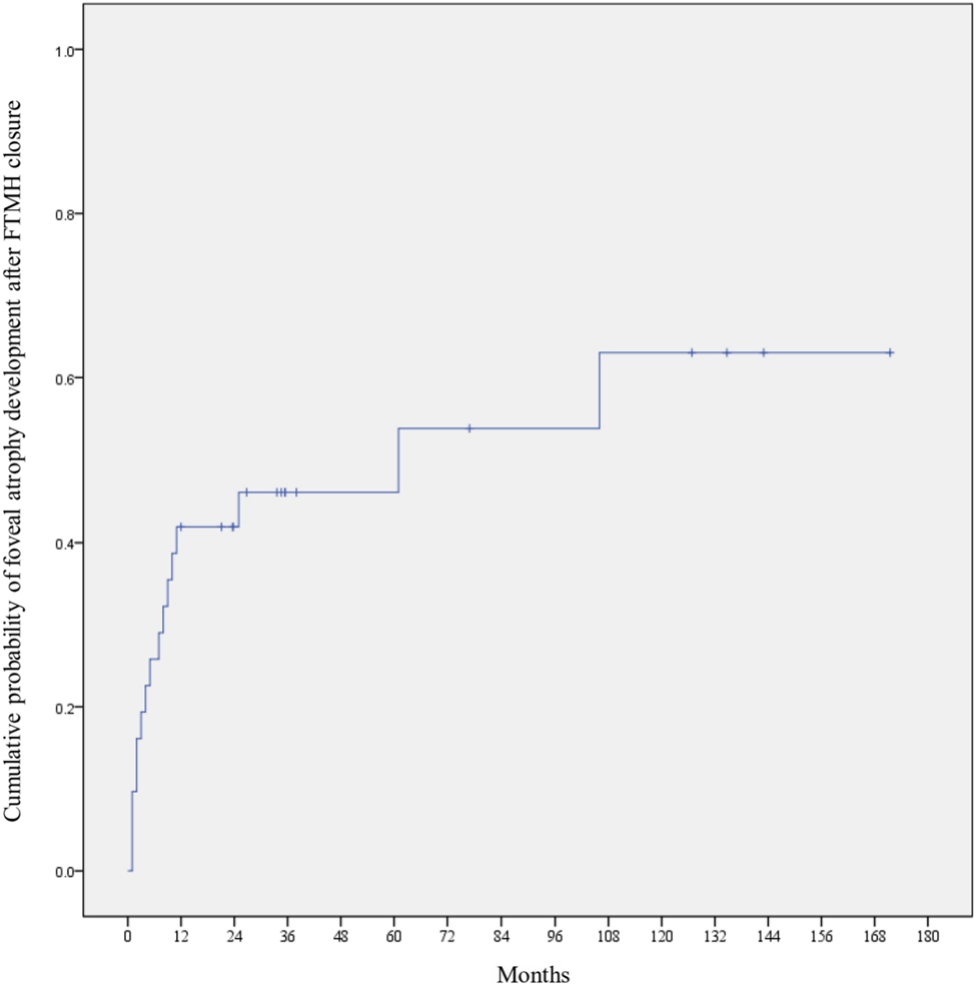
Kaplan–Meier survival curve depicting the development of foveal atrophy in patients who ultimately achieved macular hole closure after vitrectomy. The cumulative probabilities of foveal atrophy were 42.0% at 1 year and 2 years, and 46.1% at 3 and 5 years after hole closure

**Fig. 2:**
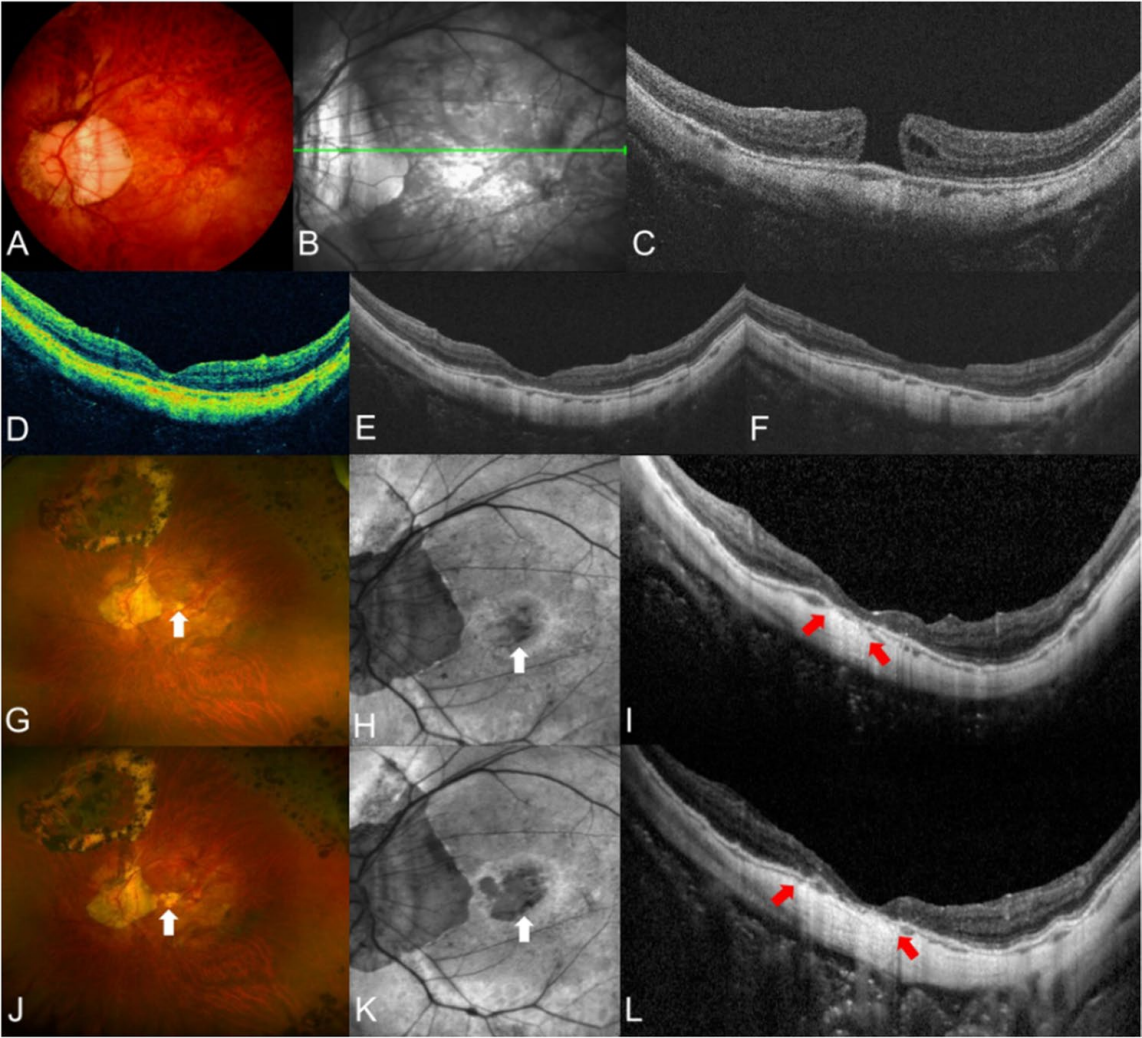
Fundus photography, near-infrared image, autofluorescence image, and optical coherence tomography (OCT) image of a 58-year-old male with a full-thickness macular hole (FTMH) in his left eye. His preoperative best-corrected visual acuity (BCVA) was 20/28, and axial length was 32.0 mm. OCT revealed an FTMH with a minimal diameter of 285.5 μm, along with intraretinal fluid and cuffing **A**, **B**, and **C**. He underwent vitrectomy with internal limiting membrane peeling and intravitreal gas tamponade. Anatomical closure was achieved post-surgery, and the macular hole did not recur (**D**, **E**, and **F**: 1 month, 1 year, and 6 years after surgery, respectively). However, foveal atrophy developed 9 years postoperatively and progressed over time (**G**, **H**, and **I**: 11 years after surgery; **J**, **K**, and **L**: 13 years after surgery). White and red arrows indicate the area of foveal atrophy. His BCVA at 1 year after surgery was 20/66 but worsened to 20/125 at the final follow-up visit, 13 years postoperatively

**Fig. 3:**
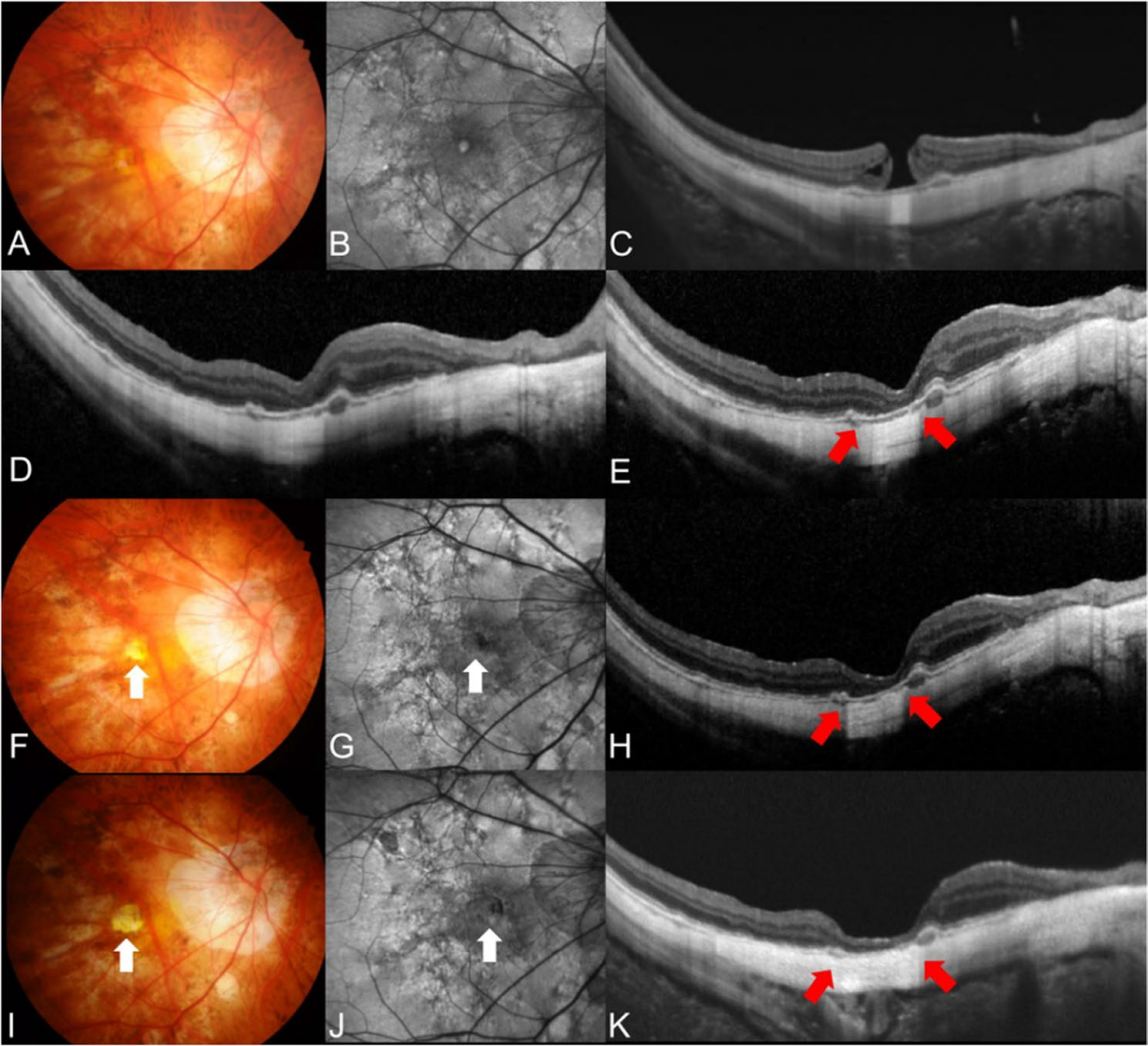
Fundus photography, autofluorescence image, and optical coherence tomography (OCT) image of a 70-year-old female with a full-thickness macular hole in her right eye. Her preoperative best-corrected visual acuity (BCVA) was 20/66, with an axial length of 31.4 mm. The minimal diameter of the macular hole was 244.0 μm **A**, **B**, and **C**. She underwent vitrectomy with internal limiting membrane peeling and intravitreal gas tamponade. Immediate postoperative OCT confirmed anatomical closure of the macular hole **D**. However, foveal atrophy developed 3 months postoperatively **E**, with further progression observed at 6 months **F**, **G**, and **H** and 2 years **I**, **J**, and **K** after surgery. Her final BCVA was 20/1000. White and red arrows indicate areas of foveal atrophy

**Fig. 4:**
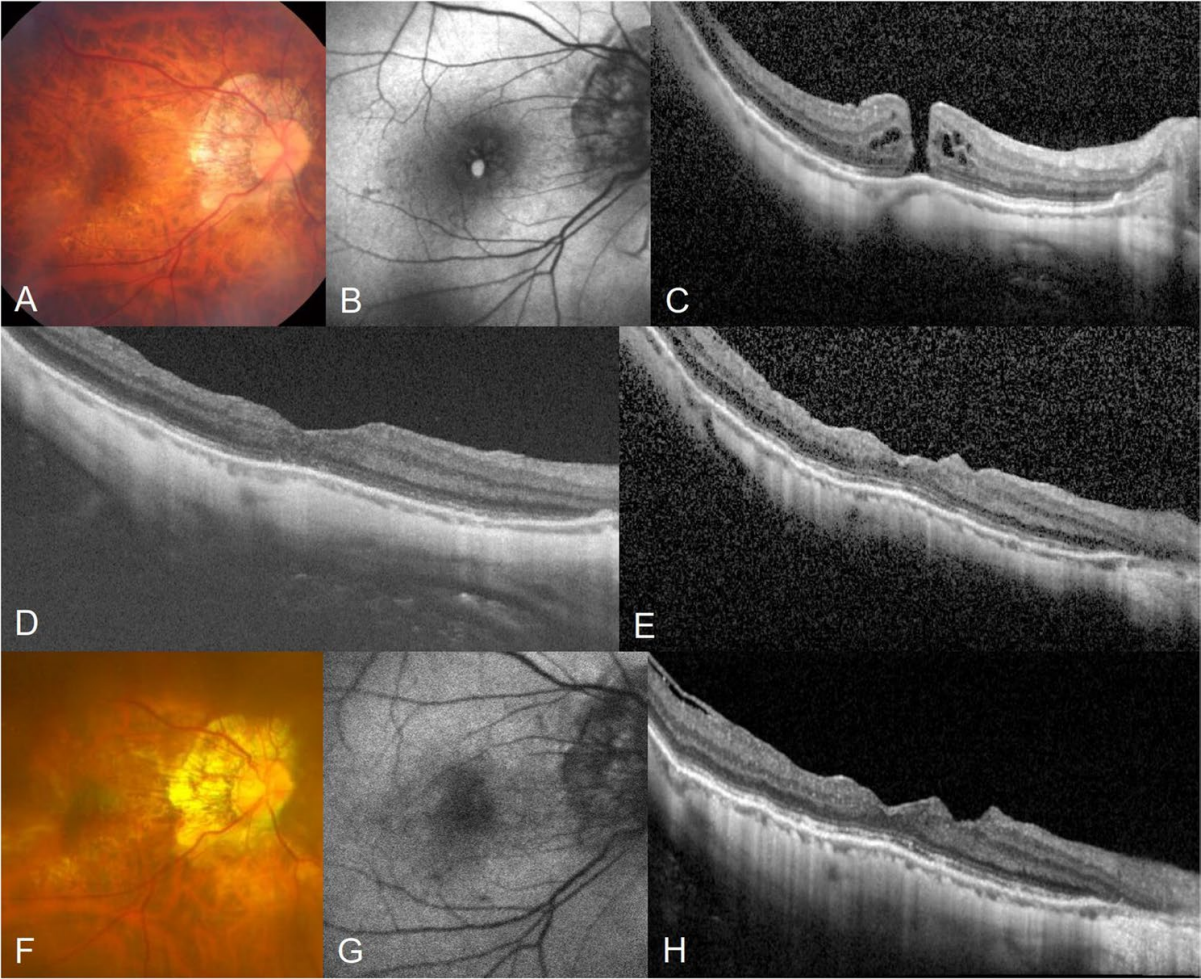
Fundus photography, autofluorescence image, and optical coherence tomography (OCT) image of a 56-year-old female with a full-thickness macular hole (FTMH) in her right eye. Her preoperative best-corrected visual acuity (BCVA) was 20/200, and axial length was 30.1 mm **A**, **B** and **C**. Duration of FTMH was 12 days, with minimal diameter and basal diameter of macular hole of 313.5 mm and 539.5 mm, respectively. She underwent vitrectomy with internal limiting membrane peeling and intravitreal gas tamponade. OCT images obtained 1 month **D** and 2 years **E** after surgery demonstrated well closed FTMH. At the final follow-up at 7 years after surgery, FTMH remained well closed without development of foveal atrophy **F**, **G** and **H**. Her final BCVA was 20/50, indicating a favorable functional outcome in conjunction with anatomical success

Primary macular hole closure was accomplished in 28 (84.8%) eyes after the first surgery. Of the five eyes with primary hole closure failure, two eyes achieved closure after a second surgery, and one eye after a third surgery. Specific techniques used in additional vitrectomies are illustrated in Table 2. Ultimately, hole closure was accomplished in 31 (93.9%) eyes, but 16 (51.6%) of them developed FA during postoperative follow-up. Thus, anatomical success was accomplished in 15 (45.5%), while anatomical failure was observed in 18 (54.5%) of 33 eyes. Among the 31 eyes that achieved final hole closure, Kaplan–Meier analysis estimated the cumulative probabilities of FA to be 42.0% at 1 year and 2 years, and 46.1% at 3 and 5 years after hole closure (Fig. 1). Univariable and multivariable Cox proportional hazard models showed that greater minimal macular hole diameter (OR: 1.064; 95% CI: 1.02–1.11; *P* = 0.002) and the existence of PS (OR: 8.66; 95% CI: 1.10–68.2; *P* = 0.040) were associated with anatomical failure (Table 3). Representative cases of macular hole closure after vitrectomy, followed by a subsequent anatomical failure due to FA, is illustrated in Figs. 2 and 3, while representative case of anatomical success is illustrated in Fig. 4.

**Table 2:** Clinical characteristics and surgical technique of additional vitrectomies of eyes that failed to close full-thickness macular hole after the initial surgery

Case	AXL (mm)	Posterior Staphyloma	State after initial surgery	Surgical technique of additional vitrectomy	Anatomical result	Final BCVA
F/77 OS	27.8	Wide macular	Unclosed FTMH	2nd Op.: repeat PPV, further ILM peeling, APC injection and SOI 3rd Op.: SO removal, repeat PPV, APC injection and IVGI (C3F8 14%)	Failure: FTMH closed, FA developed	20/200
F/76 OD	30	Wide macular	Unclosed FTMH	IVGI (C3F8 18%), APC injection without further ILM removal	Failure: FTMH closed, FA developed	20/50
F/57 OS	28.2	Absent	Unclosed FTMH	Repeat PPV, further ILM peeling, APC injection and IVGI (C3F8 15%)	Failure: FTMH closed, FA developed	20/132
F/57 OS	32.1	Wide macular	Unclosed FTMH	Additional surgery refused by patient.	Failure: FTMH remained open	20/132
F/63 OD	32.7	Wide macular	Unclosed FTMH, RD	2nd Op.: repeat PPV and SOI 3rd Op.: repeat PPV, internal SRFD, macular buckling, endolaser photocoagulation, and SOI	Failure: FTMH not closed	HM

*AXL* Axial length, *BCVA* Best corrected visual acuity, *FTMH* Full-thickness macular hole, *Op.* Operation, *PPV* Pars plana vitrectomy, *ILM* Internal limiting membrane, *APC* Autologous platelet concentrate, *SOI* Silicone oil injection, *SO* Silicone oil, *IVGI* Intravitreal gas injection, *FA* Foveal atrophy, *RD* Retinal detachment, *SRFD* Subretinal fluid drainage, *HM* Hand motion

**Table 3:** Cox proportional hazard model for variables associated with anatomical failure

Variables	Univariate analysis			Multivariate analysis		
	Odds Ratio	95% CI	*P* value	Odds Ratio	95% CI	*P* value
Axial length	1.37	1.06–1.79	0.018*			0.229
Baseline logMAR BCVA	3.02	0.80-11.45	0.104			
Macular hole duration	1.02	1.00-1.04	0.036*			0.968
ATN classification A grading						0.590
A1	[Reference]					
A2	2.53	0.51–12.57	0.257			
A3	4.28	0.93–19.80	0.063			
Dome shaped macula	2.33	0.87–6.26	0.092			0.688
Posterior staphyloma	8.12	1.06-62.00	0.044*	8.655	1.10-68.18	0.040*
Margin cystic change	0.47	0.18–1.26	0.132			
Cuff	0.98	0.22–4.37	0.975			0.431
MH minimal diameter (per 10 μm)	1.05	1.02–1.09	0.002*	1.064	1.02–1.11	0.002*
MH basal diameter (per 10 μm)	1.01	1.00-1.03	0.175			
MH height (per 10 μm)	0.93	0.87–0.99	0.032*			0.334
HFF	0.04	0.00-0.54	0.016*			0.988
THI	0.12	0.03–0.47	0.002*			0.250
MHI	0.05	0.00-0.91	0.043*			0.682
DHI	9.71	1.70-55.29	0.010*			0.246

*LogMAR* Logarithm of the minimum angle of resolution, *BCVA* Best corrected visual acuities, *MH* Macular hole, *HFF* Hole form factor, *THI* Tractional hole index, *MHI* Macular hole index, *DHI* Diameter hole index

*P < 0.05

### Functional results

**Fig. 5:**
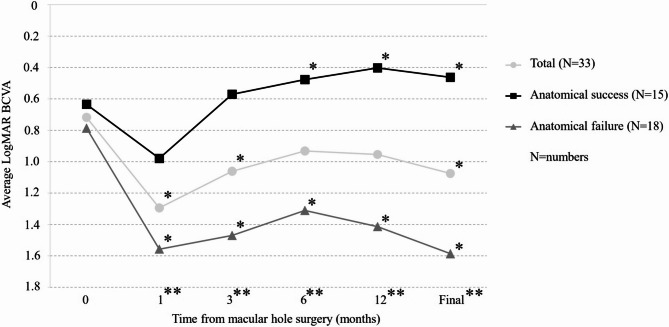
Change in mean best-corrected visual acuity during follow-up for all patients and those with and without anatomical success at final visits. A single asterisk (*) indicates a statistically significant change compared to the baseline, while double asterisks (**) indicate a time point where a significant difference occurred between the groups

**Fig. 6:**
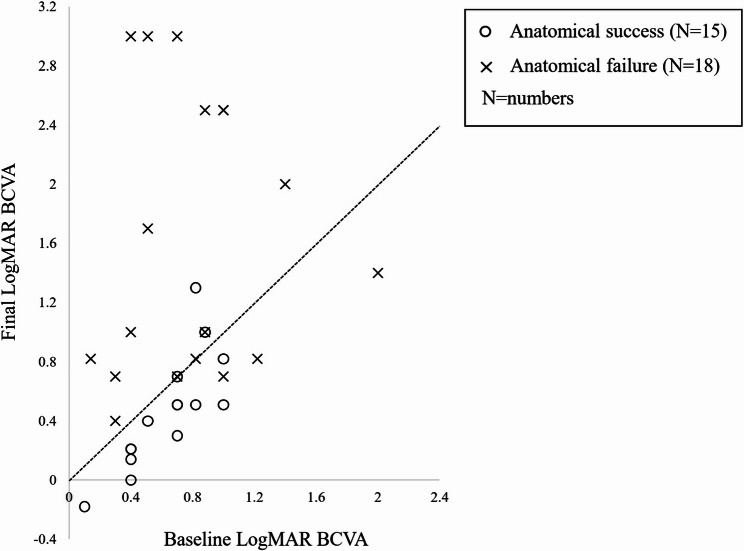
A scatterogram shows the relationship between baseline and final best-corrected visual acuity (BCVA) for each patient. The circles represent the anatomical success group, while the crosses indicate the anatomical failure group. The black dashed line represents the trendline where the baseline and final BCVA are equal. In the anatomical failure group, a statistically significant correlation was not observed between baseline and final BCVA (*P* = 0.449, Spearman’s rank correlation coefficient). However, in the anatomical success group, a statistically significant correlation was observed between baseline and final BCVA (*P* < 0.001, Spearman’s rank correlation coefficient)

Postoperative changes in BCVA during follow-up are shown in Fig. 5. Across all the eyes, mean BCVA was significantly worse than baseline at 1 month, 3 months post-surgery, and at the final visit. In contrast, mean BCVA at 6 months and 12 months post-surgery did not significantly differ from baseline. Eyes with anatomical success showed significant postoperative visual improvement from baseline at 6 months, 12 months and final visit (*P* = 0.002, *P* < 0.001, *P* = 0.0134, respectively, paired t-test), whereas those with anatomical failure showed significant deterioration in mean BCVA at all postoperative time points. Although mean baseline BCVA was comparable between eyes with anatomical success and failure, significant inter-group differences were observed at all postoperative time points (*P* ≤ 0.031, paired t-test). Figure 6 shows the distribution of preoperative and final BCVA according to anatomical results. A significantly higher proportion of eyes with anatomical success showed final BCVA improvement from baseline compared to those with anatomical failure (*n* = 12/15 [80.0%] vs. *n* = 3/18 [16.7%]; *P* = 0.001, chi-squared test). In the anatomical success group, participants with better preoperative BCVA achieved better final BCVA (*P* < 0.001, R^2^ = 0.635, Spearman’s rank correlation coefficient). In contrast, preoperative and final BCVA were not significantly correlated in the anatomical failure group (*P* = 0.449, R^2^ = 0.007, Spearman’s rank correlation coefficient). Univariable and multivariable linear regression analyses revealed that longer AXL was the only variable significantly related to worse final BCVA (*P* < 0.001; Table 4).

**Table 4:** Univariable and multivariable linear regression analysis of factors associated with final best-corrected visual acuity

Variables	Univariable analysis		Multivariable analysis	
	β	*P*	β	*P*
Axial length	0.664	< 0.001*	0.635	< 0.001*
Baseline logMAR BCVA	0.438	0.011*	0.057	0.710
Macular hole duration	0.164	0.360		
ATN classification A grading		0.017*	0.163	0.323
Dome shaped macula		0.094	0.076	0.621
Posterior staphyloma		0.004*	0.068	0.703
Margin cystic change		0.651		
Cuff		0.977		
MH minimal diameter	0.437	0.011*	0.060	0.690
MH basal diameter	0.113	0.532		
MH height	−0.546	0.001*	−0.211	0.193
HFF	−0.501	0.003*	0.053	0.712
THI	−0.611	< 0.001*	−0.147	0.374
MHI	−0.414	0.017*	0.022	0.882
DHI	0.592	< 0.001*	0.294	0.054

Kruskal Wallis test was used for univariable analysis of ATN classification A grading. Mann-Whitney U test was used for univariable analysis of dome shaped macula, cuff, margin cystic change and posterior staphyloma. Spearman’s rank correlation coefficient was used for univariable analysis of other variables. Multiple logistic regression analysis was used for multivariable analysis

*LogMAR* Logarithm of the minimum angle of resolution, *BCVA* Best corrected visual acuity, *MH* Macular hole, *HFF* Hole form factor, *THI* Tractional hole index, *MHI* Macular hole index, *DHI* Diameter hole index

*P < 0.05

### Complications

Postoperative complications were observed in four eyes. Although one patient with anatomical success developed secondary ERM during follow-up, it did not affect vision significantly as it did not involve the fovea. One eye experienced retinal detachment resulting from an unclosed FTMH, which failed to close even after two additional vitrectomies (Table 2), though the retina was successfully reattached. Two other eyes developed retinal detachment unrelated to FTMH, which had been successfully closed following the initial surgery. In these eyes, retinal detachment was successfully treated with additional vitrectomies with internal subretinal fluid drainage, endolaser photocoagulation, proliferative vitreoretinopathy membrane removal, and silicone oil tamponade. However, these eyes eventually developed FA. Thus, three cases that developed retinal detachment were all classified as the anatomical failure group, but their exclusion from inter-group BCVA comparison did not change the results, in which the anatomical success group showed statistically better BCVA across all postoperative time points (*P* ≤ 0.021, paired t-test).

## Discussion

In this study, we evaluated the surgical outcomes of highly myopic FTMH without retinoschisis. Anatomical hole closure was accomplished in 31 (93.9%) eyes, with 16 (51.6%) of those eyes later developing FA. Patients who achieved hole closure without developing FA showed vision improvement starting 6 months post-surgery, while those who experienced anatomical failure showed vision deterioration at all postoperative time points. A greater minimal macular hole diameter and the presence of PS were identified as significant risk factors for anatomical failure, and a longer AXL was the only risk factor related to worse final BCVA.

In high myopia, FTMH is commonly related to retinoschitic change, and FTMH without retinoschisis is believed to have different underlying pathogenesis, clinical course, and treatment outcomes compared to the schitic form of FTMH [17]. Li et al. [9] evaluated the clinical features of FTMH with and without macular schisis, and found that the schitic type had a longer AXL, greater myopic refractive error, and a higher incidence of PS, while BCVA was comparable between the two groups. Jo et al. [8] retrospectively evaluated surgical outcomes in FTMH with and without retinoschisis and found a better hole closure rate in the non-schitic group compared to the schitic group (78 vs. 50%), though the sample size was small. In a review of prior case series, Alkabes et al. [5] found that highly myopic FTMH without schitic change generally had a better prognosis with higher closure rates and improved functional outcomes than the schitic group. Although we did not perform a direct comparison, the closure rate of 93.9% in highly myopic FTMH without retinoschitic change in this study is comparable to the reported rates of 93–95.7% in non-myopic idiopathic FTMH in the literatures [18–20].

While the pathophysiology of schitic FTMH is relatively well understood [21, 22], the exact mechanism behind FTMH without schitic change remains uncertain. Since eyes with non-schitic FTMH are thought to be under less severe anteroposterior traction considering a lower incidence of PS and shorter AXL [9], other factors may contribute to hole formation. One possible mechanism could be the degeneration of the inner retinal layers at the foveal center, which was suggested as pathogenesis of idiopathic FTMH [23]. During the atrophic stage of mCNV, FTMH can develop with or without schitic changes at the border of old mCNV and chorioretinal atrophy [24]; however, this was not observed in the five eyes with old mCNV in the present study. Another possibility is that retinoschisis may have resolved following FTMH formation due to the release of traction by a certain reason such as disruption of ILM [25, 26]. Further investigation including serial OCT imaging is needed to elucidate the mechanism of FTMH without schitic changes in high myopia.

In our study, a longer AXL was significantly associated with poorer final BCVA. Highly myopic eyes with a longer baseline AXL have a greater likelihood of experiencing continuous and greater axial elongation during follow-up [27], which contributes to worse functional outcomes due to retinal thinning, mechanical stress, and distortion of the photoreceptor layer, although little is known regarding AXL changes after vitrectomy in highly myopic eyes. Moreover, the group that achieved anatomical hole closure demonstrated significantly better visual function than the failure group, emphasizing the importance of anatomical hole closure in non-schitic FTMH. Regarding postoperative anatomical failure, the presence of PS and a greater minimal hole diameter were identified as risk factors. In a prior study conducted by Alkabes et al. [11], minimal macular hole diameter and hole form factor were associated with postoperative BCVA in myopic macular holes without schitic change. However, the proportion of patients with DSM and PS in their study was different from that in ours, and this may have resulted in different results for OCT-based anatomical parameters. Unlike non-myopic FTMH, where hole basal diameter is considered a prognostic factor for hole closure [28, 29], our study found no association between basal diameter and hole closure, suggesting a different hole closure pattern from non-myopic FTMH.

In this study, FA development during postoperative follow-up was the primary reason for poor functional outcomes even after successful hole closure in FTMH without retinoschitic change. Among the 18 eyes classified as having anatomical failure, only 2 eyes failed to achieve hole closure following surgery, while the remaining 16 eyes developed postoperative FA despite successful closure. These findings indicate that postoperative FA was a major contributing factor to anatomical failure in this study. Similar to non-myopic eyes with idiopathic FTMH, atrophic changes in the RPE can occur postoperatively in the area of the macular hole. Multiple factors, including light toxicity, indirect mechanical injury to the RPE or photoreceptor during surgical manipulation, and retinal dimpling caused by ILM peeling, have been proposed as risk factors for FA development [10, 30, 31]. Engelbrecht et al. [32] reported that incidence of RPE change following macular hole operation was 47.6%, emphasizing the toxic influence of ICG used during surgery to facilitate visualization of ILM. Intraoperative use of ICG, which can potentially come into direct contact with the exposed RPE at the site of FTMH, can shorten the “safe time” for light exposure to the macula, increasing phototoxicity to the RPE. In highly myopic eyes, Fang et al. [10] observed that the fovea-centered macular atrophy developed in 10.5% following vitrectomy for MTM. The risk was higher in eyes with FTMH or macular hole retinal detachment than in those with only myopic retinoschisis, suggesting that direct exposure of the RPE may be a major risk factor for FA in high myopia. Incidence of FA in our study is higher than in the study by Fang et al., and the difference may be attributed to longer follow-up period in this study and potentially different pathogenesis between FTMH without retinoschisis and MTM in highly myopic eyes.

In highly myopic patients, long-term accumulation of residual ICG following PPV with ICG-assisted ILM removal is more pronounced in eyes having moderate or severe myopic chorioretinal atrophy than in those without [33]. This suggests that RPE status may be associated with ICG clearance and accumulation. Otherwise, RPE and retina of highly myopic eyes may be inherently more susceptible to potential phototoxicity caused by remnant ICG than non-myopic eyes. In high myopia patients, especially those with PS, surgical maneuvers such as removal of remnant vitreous cortex overlying the retina and peeling of ILM are more challenging and time-consuming, causing greater mechanical trauma and light-induced damage to the retina and RPE due to the increased AXL, more concave surgical plane, thinner retina, and reduced contrast caused by underlying chorioretinal atrophy [34, 35]. Furthermore, unlike non-myopic eyes, progressive scleral ectasia and axial elongation would exert chronic mechanical stress on the RPE. Therefore, due to the intrinsic features of highly myopic eyes, FTMH without retinoschitic changes in high myopia is at a higher risk of occurring FA postoperatively compared to idiopathic FTMH in non-myopic patients despite having a comparable configuration.

Among the five eyes that initially failed to achieve anatomical hole closure, three underwent repeated vitrectomy with the use of autologous platelet concentrate (APC), which harbors multiple growth factors that facilitate cellular regeneration and tissue repair. It also helps migration and cellular proliferation of Müller cells [36, 37]. In a randomized controlled trial involving recurrent macular holes, large macular holes, or high myopia macular holes [37], the use of APC in conjunction with PPV resulted in a greater hole closure rate compared to PPV alone, but the difference did not reach statistical significance. In this study, although hole closure was achieved in all three eyes, FA eventually developed in each case. This suggests that APC may be effective for the closure of recalcitrant non-schitic FTMH in high myopia, but it does not prevent development of FA. In these eyes, the risk of FA may have been increased due to heightened stress on the RPE during repeated surgeries, as well as pro-inflammatory cytokines contained in the APC [38], which may promote cellular apoptosis. Further investigation is warranted to elucidate the potential association between APC and the development of FA.

This study has some limitations, including the nature of a retrospective study. First, five surgeons were included in our study, and variations in surgical techniques according to different surgeons could have influenced the surgical outcomes. Also, though inverted ILM flap technique has currently become a standard approach in treating FTMH in highly myopic eyes, there was no clear consensus on its use during the earlier years of our study period. Therefore, none of the patients underwent the inverted ILM flap technique in this study. Second, this study included only 33 eyes; however, by applying strict inclusion criteria, we could form a homogenous patient group that had FTMH without retinoschisis. Third, the follow-up period varied among patients, which may limit the accurate assessment of the incidence and risk factors of long-term FA. Fourth, since our aim was to evaluate comprehensive postoperative outcomes, we considered either case with postoperative FA or unclosed macular holes as clinically important components of anatomical failure. Further analysis is needed to investigate the factors associated with the development of FA specifically among patients who achieved macular hole closure. Finally, the treatment outcomes of highly myopic non-schitic FTMH were not compared with those of schitic FTMH in high myopia or non-schitic idiopathic FTMH in non-myopic eyes.

In conclusion, the hole closure rate after vitrectomy for high myopic FTMH without retinoschitic change was high, but the development of postoperative FA limited vision improvement. The presence of PS and a greater minimal hole diameter were associated with anatomical failure, while the longer AXL was associated with worse functional outcomes. Further studies, including serial OCT imaging during the development of hole would elucidate the pathogenesis of FTMH without retinoschitic change in patients with high myopia.

## Data Availability

The datasets analyzed during the current study are available from the corresponding author on reasonable request.
